# Association of subcutaneous and visceral adipose tissue with overall survival in Taiwanese patients with bone metastases – results from a retrospective analysis of consecutively collected data

**DOI:** 10.1371/journal.pone.0228360

**Published:** 2020-01-30

**Authors:** Wen Ching Chuang, Ngan Ming Tsang, Chi Cheng Chuang, Kai Ping Chang, Ping Ching Pai, Kuan Hung Chen, Wen Chi Chou, Shiao Fwu Tai, Shu Chen Liu, Kin Fong Lei

**Affiliations:** 1 Chang Gung University, Medicine, Taoyuan, Taiwan; 2 Chang Gung Memorial Hospital, Linkou Branch, Taoyuan, Taiwan; 3 Department of Radiation Oncology, Linkou Chang Gung Memorial Hospital and Chang Gung University, Taoyuan, Taiwan; 4 School of Traditional Chinese Medicine, Chang Gung University, Taoyuan, Taiwan; 5 Department of Neurosurgery, Chang Gung Memorial Hospital and University at Lin-Kou, Taoyuan, Taiwan; 6 Department of Otolaryngology-Head Neck Surgery, Linkou Chang Gung Memorial Hospital and Chang Gung University at Lin-Kou, Taoyuan, Taiwan; 7 Division of Hematology-Oncology, Department of Internal Medicine, Chang Gung Memorial Hospital Linkou Branch, and School of Medicine, Chang Gung, Taoyuan, Taiwan; 8 Department of Otorhinolaryngology, Chang Gung Memorial Hospital, Linkou Branch, Taoyuan, Taiwan; 9 Department of Biomedical Sciences and Engineering, National Central University, Taoyuan, Taiwan; 10 Graduate Institute of Biomedical Engineering, Chang Gung University, Taoyuan, Taiwan; San Raffaele Roma Open University, ITALY

## Abstract

**Background:**

Growing evidence indicates that measures of body composition may be related to clinical outcomes in patients with malignancies. The aim of this study was to investigate whether measures of regional adiposity–including subcutaneous adipose tissue index (SATI) and visceral adipose tissue index (VATI)–can be associated with overall survival (OS) in Taiwanese patients with bone metastases.

**Methods:**

This is a retrospective analysis of prospectively collected data. We examined 1280 patients with bone metastases who had undergone radiotherapy (RT) between March 2005 and August 2013. Body composition (SATI, VATI, and muscle index) was assessed by computed tomography at the third lumbar vertebra and normalized for patient height. Patients were divided into low- and high-adiposity groups (for both SATI and VATI) according to sex-specific median values.

**Results:**

Both SATI (hazard ratio [HR]: 0.696; P<0.001) and VATI (HR: 0.87; P = 0.037)–but not muscle index–were independently associated with a more favorable OS, with the former showing a stronger relationship. The most favorable OS was observed in women with high SATI (11.21 months; 95% confidence interval: 9.434−12.988; P<0.001).

**Conclusions:**

High SATI and VATI are associated with a more favorable OS in Taiwanese patients with bone metastases referred for RT. The question as to whether clinical measures aimed at improving adiposity may improve OS in this clinical population deserves further scrutiny.

## Introduction

Bone represents one of the most common sites for cancer spread, especially in patients with breast, prostate, or lung malignancies.[1, 2] Bone metastases are a significant source of morbidity, decreased performance status, and impaired quality of life. Moreover, the presence of bone metastases typically portends a poor prognosis, with a median overall survival (OS) of 6−7 months.[3]

Several factors–including clinical stage, patient demographics, and tumor histology–have been shown to affect the OS of patients with bone metastases.[4] Notably, sex disparities have been reported in the survival of patients with metastatic spread to the bone–with mortality rate ratios being significantly higher in males than in females for most malignancies.[5] Women also have a higher total adiposity than men, with a preponderance of subcutaneous adipose tissue. In contrast, men typically tend to accumulate visceral adipose tissue.[6] Subcutaneous and visceral adipose tissue indices (SATI and VATI, respectively) may influence the clinical outcomes of patients with cancer in a sex-dependent manner. [7]

Previous studies have reported a significant prognostic impact of SATI and VATI in different solid tumors, including advanced renal cell carcinoma, hepatocellular carcinoma, and pancreatic cancer,[8–10] although there has been some discrepant findings and the therapeutic implications of these observations have not been fully elucidated [11].

The aim of this study was to investigate whether measures of regional adiposity–including SATI and VATI–can be associated with overall survival (OS) in Taiwanese patients with bone metastases who were referred for radiotherapy (RT).

## Materials and methods

### Study patients

The present study was designed as a retrospective review of prospectively collected data and was conducted in a radiation oncology setting. Between March 2005 and August 2013, a total of 1654 Taiwanese patients with bone metastases were consecutively referred for RT to the Chang Gung Memorial Hospital. All of them had a histology-proven diagnosis of cancer and underwent computed tomography (CT) imaging within 30 days of the initial assessment. The diagnosis of bone metastases was based on the results of bone scintigraphy, X-ray, CT, or magnetic resonance imaging. Patients were excluded in presence of the following criteria: age <18 years, unavailable CT scans within two weeks before the start of RT, or lack of measures of weight and height within two weeks of enrolment. A total of 374 cases met the exclusion criteria, resulting in a final study sample of 1280 patients. The study protocol was reviewed and approved by the Institution Review Board of the Chang Gung Memorial Hospital (approval number: IRB: 201701224B0). Owing to the retrospective nature of the analysis, the need for informed consent was waived. Data collection from electronic medical records was supervised by an experienced nurse and a radiation oncologist.

### CT-based body composition analysis

In keeping with previous methodology,[12] single-slice CT imaging at level L3 was used to analyze adiposity. SATI and VATI were identified according to Hounsfield units (HU) (from -190 to -30 HU for SATI and from -29 to 150 HU for VATI, respectively). The tissue cross-sectional areas (expressed in cm^2^) were calculated automatically by the CT software after normalization for patient height. SATI, VATI, total adipose tissue, and skeletal muscle indexes were expressed in cm^2^ m^-2^. All adiposity measures were taken in the two weeks preceding the start of RT.

### Variable definition

Owing to the lack of a commonly accepted standard, SATI, VATI, and skeletal muscle indices were dichotomized according to median values measured at L3. OS was defined as the time elapsed from the start of RT for bone metastases to the date of death. Body mass index (BMI) was categorized as follows: underweight (BMI <18.5 kg/m^2^), normal weight (BMI: 18.5–24.99 kg/m^2^), overweight (BMI: 25–29.99 kg/m^2^), and obese (BMI ≥30 kg/m^2^). Equivalent doses in 2-Gy fraction (EQD2Gy) were used to express different total radiation doses in terms of amount and number of fractions. The time to metastases (calculated from the time of diagnosis of primary cancer to the identification of distant metastases) was categorized in ≤ 1 year *versus* >1 year. Metastases were considered multiple in presence of simultaneous involvement of at least two organs or different parts of skeleton (e.g., sternum and sacrum). The use of systemic therapy was investigated in the timeframe ranging from 1 month before RT to the date of the last follow-up. Other variables of interest were previously described.[13] The presence of comorbidities was dichotomized (yes *versus* no) according to the Charlson comorbidity index. Employment status was classified into three categories using the Registrar General’s Social Class (RGSC) scheme, as follows: unemployed, low-wage employed, and high-wage employed. Education status was categorized as high *versus* low (junior high school and above *versus* elementary school and below). The patient’s place of residence was dichotomized as either rural or urban (population density below or above 800 persons per km^2^, respectively). Risky oral habits were classified as follows: cigarette smoking (smoked ≥100 in lifetime *versus* < 100 cigarettes in lifetime and no current smoking), betel quid chewing (current/former *versus* never), and alcohol drinking (current/former *versus* never).

### Statistical analysis

Continuous variables were compared using the Student’s *t*-test, whereas the Pearson’s chi-square test was used for categorical variables. The associations between the study variables (including indices of adiposity) and OS were investigated using univariate and multivariate Cox proportional hazard ratio analyses. Results were expressed as hazard ratios (HRs) with their 95% confidence interval (CIs). We also categorized patients according to SATI and VATI values (high *versus* low, with high values serving as references). Survival plots were constructed with the Kaplan-Meier method (log-rank test).Two-tailed P values <0.05 were considered statistically significant. Owing to the exploratory nature of the study, the Bonferroni’s correction was not applied.

## Results

### Patient characteristics

The general characteristics of the study patients are summarized in Table 1. Of the 1280 participants, 1237 were followed up until death, whereas the remaining 43 were censored on the date last known to be alive. The study cohort included 740 (57.8%) men and 540 (42.2%) women. The most common primary cancer site was the lung (35% in both sexes), and there were 897 (70%) patients with an ECOG performance status of 0−1. The interval between the diagnosis of primary cancer and the detection of metastases was 0.11 months in women (95% CI: 0−15.64 months) and 0.04 months (95% CI: 0−13.50 months) in men, respectively. Table 1 shows the results pertaining to adiposity indices. Men had higher skeletal muscle and VATI than women, whereas SATI was higher in women.

**Table 1:** Patient characteristics according to the subcutaneous and visceral adiposity status.

		SATI				VATI			
		Low	High	Total number	P value	Low	High	Total number	P value
**Number of patients**		640 (50.0%)	640 (50.0%)	1280 (100%)		640 (50.0%)	640 (50.0%)	1280 (100%)	
**SATI**									
	Low					455 (71.1%)	185 (28.9%)	640 (50.0%)	<0.001^a^
	High					185 (28.9%)	455 (71.1%)	640 (50.0%)	
	Median	8.15 (0.01–15.48)	27.77 (15.50–148.77)	15.49 (0.01–148.77)	<0.001^b^	9.93 (0.01–71.92)	23.30 (1.24–148.77)	15.49 (0.01–148.77)	<0.001^b^
	Mean ± SD, cm^2^/m^2^	7.96±4.58	33.42±17.87	20.69±18.22		12.81±11.92	28.57±19.96	20.69±18.22	
**VATI**									
	Low	455 (71.1%)	185 (28.9%)	640 (50.0%)	<0.001^a^				
	High	185 (28.9%)	455 (71.1%)	640 (50.05%)					
	Median	4.74 (0.02–72.53)	15.59 (0.40–93.34)	9.84 (0.02–93.34)	<0.001^b^	3.70 (0.02–9.83)	19.35 (9.84–93.34)	9.84 (0.02–93.34)	<0.001^b^
	Mean	8.10±9.39	18.35±13.19	13.23±12.54		4.07±2.92	22.39±11.75	13.23±12.54	
**Muscle index**									
	Low	293 (45.8%)	347 (54.2%)	640 (50.0%)	0.003^a^	333 (52.0%)	307 (48.0%)	640 (50.0%)	0.162^a^
	High	347 (54.2%)	293 (45.8%)	640 (50.0%)		307 (48.0%)	333 (52.0%)	640 (50.0%)	
	Median	17.23 (4.02–49.49)	16.01 (6.44–93.90)	16.61 (4.02–93.90)	0.015^b^	16.41 (4.02–43.93)	16.89 (6.44–93.90)	16.61 (4.02–93.9)	0.063^b^
	Mean	17.78±5.59	16.96±6.28	17.37±5.95		17.06±5.65	17.68±6.23	17.37±5.95	
**Age group, years**									
	<59.5	318 (49.7%)	313 (48.9%)	631 (49.3%)	0.823^a^	376 (58.8%)	255 (39.8%)	631 (49.3%)	<0.001^a^
	≥59.5	322 (50.3%)	327 (51.1%)	649 (50.7%)		264 (41.3%)	385 (60.2%)	649 (50.7%)	
	Median	59.67 (19.54–95.57)	59.83 (21.98–87.05)	59.73 (19.54–95.57)	0.503^b^	56. 79 (19.54–95.57)	63.01 (27.9–90.45)	59.73 (19.54–95.57)	<0.001^b^
	Mean	60.55±13.16	60.08±12.11	60.32±12.64		57.59±12.94	63.04±11.73	60.32±12.64	
**Sex**									
	Female	152 (23.8%)	388 (60.6%)	540 (42.2%)	<0.001^a^	287 (44.8%)	253 (39.5%)	540 (42.2%)	0.062^a^
	Male	488 (76.3%)	252 (39.4%)	740 (57.8%)		353 (55.2%)	387 (60.5%)	740 (57.8%)	
**Performance status**									
	ECOG 0–1	428 (66.9%)	469 (73.3%)	897 (70.1%)	0.015^a^	444 (69.4%)	453 (70.8%)	897 (70.1%)	0.625^a^
	ECOG 2–4	212 (33.1%)	171 (26.7%)	383 (29.9%)		196 (30.6%)	187 (29.2%)	383 (29.9%)	
**Onset of metastasis**									
	≤ 1 year	470 (73.4%)	430 (67.2%)	900 (70.3%)	0.017^a^	455 (71.1%)	445 (69.5%)	900 (70.3%)	0.541^a^
	> 1 years	170 (26.6%)	210 (32.8%)	380 (29.7%)		185 (28.9%)	195 (30/5%)	380 (29.7%)	
	Median	0.08 (0–13.50)	0.04 (0–15.64)	0.05 (0–15.64)	0.006^b^	0.08 (0–15.64)	0.04 (0–15.64)	0.05 (0–15.64)	0.652^b^
	Mean	1.04±2.03	1.40±2.56	1.22±2.32		1.19±2.28	1.25±2.36	1.22±2.32	
**Site of metastasis**									
	Bone	543 (84.8%)	542 (84.7%)	1085 (84.8%)	0.943	524 (81.9%)	561 (87.7%)	1085 (84.8%)	0.007
	Brain	17 (2.7%)	19 (3.0%)	36 (2.8%)		25 (3.9%)	11 (1.7%)	36 (2.8%)	
	Others	80 (12.5%)	79 (12.3%)	159 (12.4%)		91 (14.2%)	68 (10.6%)	159 (12.4%)	
**Multiple metastases**									
	No	121 (18.9%)	124 (19.4%)	245 (19.1%)	0.832^a^	120 (18.8%)	125 (19.5%)	245 (19.1%)	0.776^a^
	Yes	519 (81.1%)	516 (80.6%)	1035 (80.9%)		520 (81.3%)	515 (80.5%)	1035 (80.9%)	
**Site of primary cancer**									
	Lung cancer	253 (39.5%)	222 (34.7%)	475 (37.1%)	<0.001^a^	232 (36.3%)	243 (38.0%)	475 (37.1%)	<0.001^a^
	Hepatoma	75 (11.7%)	60 (9.4%)	135 (10.5%)		75 (11.7%)	60 (9.4%)	135 (10.5%)	
	Breast cancer	26 (4.1%)	90 (14.1%)	116 (9.1%)		56 (8.8%)	60 (9.4%)	116 (9.1%)	
	Prostate cancer	39 (6.1%)	53 (8.3%)	92 (7.2%)		21 (3.3%)	71 (11.1%)	92 (7.2%)	
	Rectal cancer	34 (5.3%)	43 (6.7%)	77 (6.0%)		40 (6.3%)	37 (5.8%)	77 (6.0%)	
	Others	213 (33.3%)	172 (26.9%)	385 (30.1%)		216 (33.8%)	169 (26.4%)	385 (30.1%)	
**EQD**_**2Gy**_									
	<32.5	337 (52.7%)	279 (43.6%)	616 (48.1%)	0.001^a^	325 (50.8%)	291 (45.5%)	616 (48.1%)	0.065^a^
	≥32.5	303 (47.3%)	361 (56.4%)	664 (51.9%)		315 (49.2%)	349 (54.5%)	664 (51.9%)	
	Median	31.25 (1.44–70.00)	32.50 (3.25–84.00)	32.50 (1.44–84.00)	<0.001^b^	31.98 (1.83–70.00)	32.50 (1.44–84.00)	32.50 (1.44–84.00)	0.484^b^
	Mean	28.14±10.99	30.66±10.70	29.40±10.91		29.19±11.29	29.62±10.52	29.40±10.91	
**Systemic therapy**									
	No	266 (41.6%)	164 (25.6%)	430 (33.6%)	<0.001^a^	238 (37.2%)	192 (30.0%)	430 (33.6%)	0.008^a^
	Yes	374 (58.4%)	476 (74.4%)	850 (66.4%)		402 (62.8%)	448 (70.0%)	850 (66.4%)	
**Comorbidities**									
	No	296 (46.3%)	256 (40.0%)	552 (43.1%)	0.028^a^	328 (51.2%)	224 (35.0%)	552 (43.1%)	<0.001^a^
	Yes	344 (53.8%)	384 (60.0%)	728 (56.9%)		312 (48.8%)	416 (65.0%)	728 (56.9%)	
**Employment status**									
	High	152 (23.8%)	142 (22.2%)	294 (23.0%)	<0.001^a^	152 (23.8%)	142 (22.2%)	294 (23.0%)	0.152^a^
	Low	275 (43.0%)	169 (26.4%)	444 (34.7%)		234 (36.6%)	210 (32.8%)	444 (34.7%)	
	None	213 (33.3%)	329 (51.4%)	542 (42.3%)		254 (39.7%)	288 (45.0%)	542 (42.3%)	
**Education level**									
	None/primary school	311 (48.6%)	344 (53.8%)	655 (51.2%)	0.074^a^	291 (45.5%)	364 (56.9%)	655 (51.2%)	<0.001^a^
	High school	329 (51.4%)	296 (46.3%)	625 (48.8%)		349 (54.5%)	276 (43.1%)	625 (48.8%)	
**Place of residence**									
	Urban	351 (54.8%)	345 (53.9%)	696 (54.4%)	0.779^a^	364 (56.9%)	332 (51.9%)	696 (54.4%)	0.082^a^
	Rural	289 (45.2%)	295 (46.1%)	584 (45.6%)		276 (43.1%)	308 (48.1%)	584 (45.6%)	
**Cigarette smoking**									
	No	270 (42.2%)	455 (71.1%)	725(56.6%)	<0.001^a^	356 (55.6%)	369 (57.7%)	725 (56.6%)	0.499^a^
	Yes	370 (57.8%)	185(28.9%)	555 (43.4%)		284 (44.4%)	271 (42.3%)	555 (43.4%)	
**Betel quid chewing**									
	No	511 (79.8%)	578 (90.3%)	1089 (85.1%)	<0.001^a^	538 (84.1%)	551 (86.1%)	1089 (85.1%)	0.347^a^
	Yes	129 (20.2%)	62 (9.7%)	191 (14.9%)		102 (15.9%)	89 (13.9%)	191 (14.9%)	
**Alcohol drinking**									
	No	420 (65.6%)	531 (83.0%)	951 (74.3%)	<0.001^a^	468 (73.1%)	483 (75.5%)	951 (74.3%)	0.338^a^
	Yes	220 (34.4%)	109 (17.0%)	329 (25.7%)		172 (26.9%)	157 (24.5%)	329 (25.7%)	
**Days of metastases treatment**									
	≤12	360 (56.3%)	311 (48.6%)	671 (52.4%)	0.007^a^	328 (51.2%)	343 (53.6%)	671 (52.4%)	0.433^b^
	≥13	280 (43.8%)	329 (51.4%)	609 (47.6%)		312 (48.8%)	297 (46.4%)	609 (47.6%)	
	Median	11.50 (1–93)	13.00(1–67)	12.00(1–93)	<0.001^b^	12.00 (1–93)	12.00(1–67)	12.00(1–93)	0.984^b^
	Mean	11.60±7.98	13.59 ±8.86	12.59±8.49		12.59±8.91	12.60±8.05	12.59±8.49	
**Metastasis treatment period**									
	≤2009	331 (51.7%)	298 (46.6%)	629 (49.1%)	0.074^a^	312 (48.8%)	317 (49.5%)	629 (49.1%)	0.823^a^
	≥2010	309 (48.3%)	342 (53.4%)	651 (50.9%)		328 (51.2%)	323 (50.5)	651 (50.9%)	
**Body mass index, kg/m**^**2**^									
	Underweight	116 (18.1%)	4 (0.6%)	120 (9.4%)	<0.001^a^	118 (18.4%)	2 (0.3%)	120 (9.4%)	<0.001^a^
	Normal weight	478 (74.7%)	344 (53.8%)	822 (64.2%)		465 (72.7%)	357 (55.8%)	822 (64.2%)	
	Overweight	45 (7.0%)	242 (37.8%)	287 (22.4%)		55 (8.6%)	232 (36.3%)	287 (22.4%)	
	Obese	1 (0.2%)	50 (7.9%)	51 (4.0%)		2 (0.3%)	49 (7.7%)	51 (4.0%)	
	Median	21.01 (13.34–20.65)	24.78 (16.98–38.73)	22.86 (13.34–38.73)	<0.001^b^	21.01 (13.34–30.65)	24.68 (16.98–38.73)	22.86 (13.34–38.73)	<0.001^b^
	Mean	21.12±2.74	25.08±3.32	23.10±3.63		21.12±2.74	25.08±3.32	23.10± 3.63	

Abbreviations: SD, standard deviation; ECOG, Eastern Cooperative Oncology Group; EQD2 Gy, equivalent doses in 2-Gy fractions; SATI, subcutaneous adipose tissue index; VATI, visceral adipose tissue index.

^a^Chi-square test

^b^ANOVA test

### Survival analysis

The median follow-up time for the 43 surviving patients was 78.28 months (range: 0.789−147.25 months). The median OS after RT was 6.03 months (range: 0.03−147.25 months). The 6-, 12-, 24-, and 48-month OS rates in women and men were 41.4%/61.8%, 23.6% /43.2%, 9.8%/22.6%, and 3.8%/11.2%, respectively. The median OS was 9.53 months (range: 0.10−137.42 months) in women and 4.7 months (range: 0.30−147.25) in men.

SATI values ≥11.63 cm^2^ in men and ≥25.21 cm^2^ in women were considered as high. Similarly, VATI values ≥10.46 cm^2^ in men and ≥8.96 cm^2^ in women were regarded as elevated. The median OS in the high and low SATI groups was 27.77 months (range: 15.50−148.77 months) and 8.15 months (range: 0.01−15.48 months), respectively. The median OS in the high and low VATI groups was 19.35 months (range: 9.84−93.34 months) and 3.70 months (range: 0.02−9.83 months), respectively.

The results of univariate and multivariate analyses are presented in Table 2. The following variables were independently associated with OS in multivariate analysis: SATI, VATI, sex, performance status, primary tumor site, more than one metastatic site, ECOG performance status, EQD2Gy, systemic therapy, education, days of metastases treatment, and time to metastases (Table 2).

**Table 2:** Univariate and multivariate analysis of overall survival.

		Univariate analysis		Multivariable analysis	
		Overall survival		Overall survival	
Number of patients = 1280		HR (95% CI)	P value	HR (95% CI)	P value
SATI (high *versus* low)		0.551 (0.492–0.618)	<0.001	0.696 (0.606–0.800)	<0.001
VATI (high *versus* low)		0.756 (0.676–0.846)	<0.001	0.870 (0.764–0.992)	0.037
Muscle index (high *versus* low)		1.042 (0.932–1.165)	0.470		
Age group (≥59.5 *versus* <59.5 years)		1.186 (1.061–1.327)	0.003	0.972 (0.848–1.113)	0.679
Sex (male *versus* female)		1.579 (1.408–1.770)	<0.001	1.186 (1.010–1.393)	0.037
ECOG performance status (2–4 *versus* 0–1)		1.257 (1.113–1.420)	<0.001	1.305 (1.153–1.478)	<0.001
Multiple metastases (yes *versus* no)		1.414 (1.223–1.635)	<0.001	1.350 (1.165–1.565)	<0.001
Site of primary cancer (lung *versus* other sites)		0.816 (0.727–0.916)	0.001	0.813 (0.716–0.922)	0.001
EQD_2Gy_ (≥32.5 *versus* <32.5)		0.651 (0.582–0.729)	<0.001	0.802 (0.695–0.925)	0.002
Systemic therapy (yes *versus* no)		0.584 (0.519–0.658)	<0.001	0.621 (0.546–0.706)	<0.001
Comorbidities (yes *versus* no)		1.120 (1.000–1.253)	0.049	1.065 (0.948–1.198)	0.288
Education level (high school *versus* none/primary school)		0.864 (0.772–0.966)	0.010	0.869 (0.763–0.989)	0.033
Cigarette smoking (yes *versus* no)		1.432 (1.279–1.603)	<0.001	1.092 (0.934–1.276)	0.272
Betel quid chewing (yes *versus* no)		1.331 (1.139–1.555)	<0.001	0.964 (0.804–1.155)	0.689
Alcohol drinking (yes *versus* no)		1.373 (1.209–1.560)	<0.001	1.148 (0.989–1.332)	0.069
Days of metastasis treatment (≥13 *versus* ≤12)		0.704 (0.629–0.787)	<0.001	0.864 (0.750–0.996)	0.044
Onset of metastasis (>1 year *versus* ≤1 year)		0.800 (0.708–0.904)	<0.001	0.913 (0.801–1.041)	0.175
Place of residence (urban *versus* rural)		0.985 (0.881–1.102)	0.795		
Site of metastasis			0.216		
	Bone	1.340 (0.957–1.877)	0.089		
	Brain	0.974 (0.821–1.156)	0.765		
Employment status			0.556		
(low *versus* high)		1.084 (0.932–1.260)	0.294		
(none *versus* high)		1.034 (0.895–1.195)	0.652		
Metastases treatment period (≥2010 *versus* ≤2009)		0.990 (0.886–1.107)	0.866		
Body mass index (>25 *versus* ≤25 kg/m^2^)		0.755 (0.664–0.857)	<0.001		

Abbreviations: HR, hazard ratio; CI, confidence interval; ECOG, Eastern Cooperative Oncology Group; EQD2 Gy, equivalent doses in 2-Gy fractions; SATI, subcutaneous adipose tissue index; VATI, visceral adipose tissue index. An L3 subcutaneous adipose tissue index ≥11.63 cm^2^ m^-2^ in males and ≥25.21 cm^2^ m^-2^ in females was considered as high. An L3 visceral adipose tissue index ≥10.46 cm^2^ m^-2^ in males and ≥8.96 cm^2^ m^-2^ in females was considered as high.

### Prognostic significance of SATI and VATI

**Fig 1:**
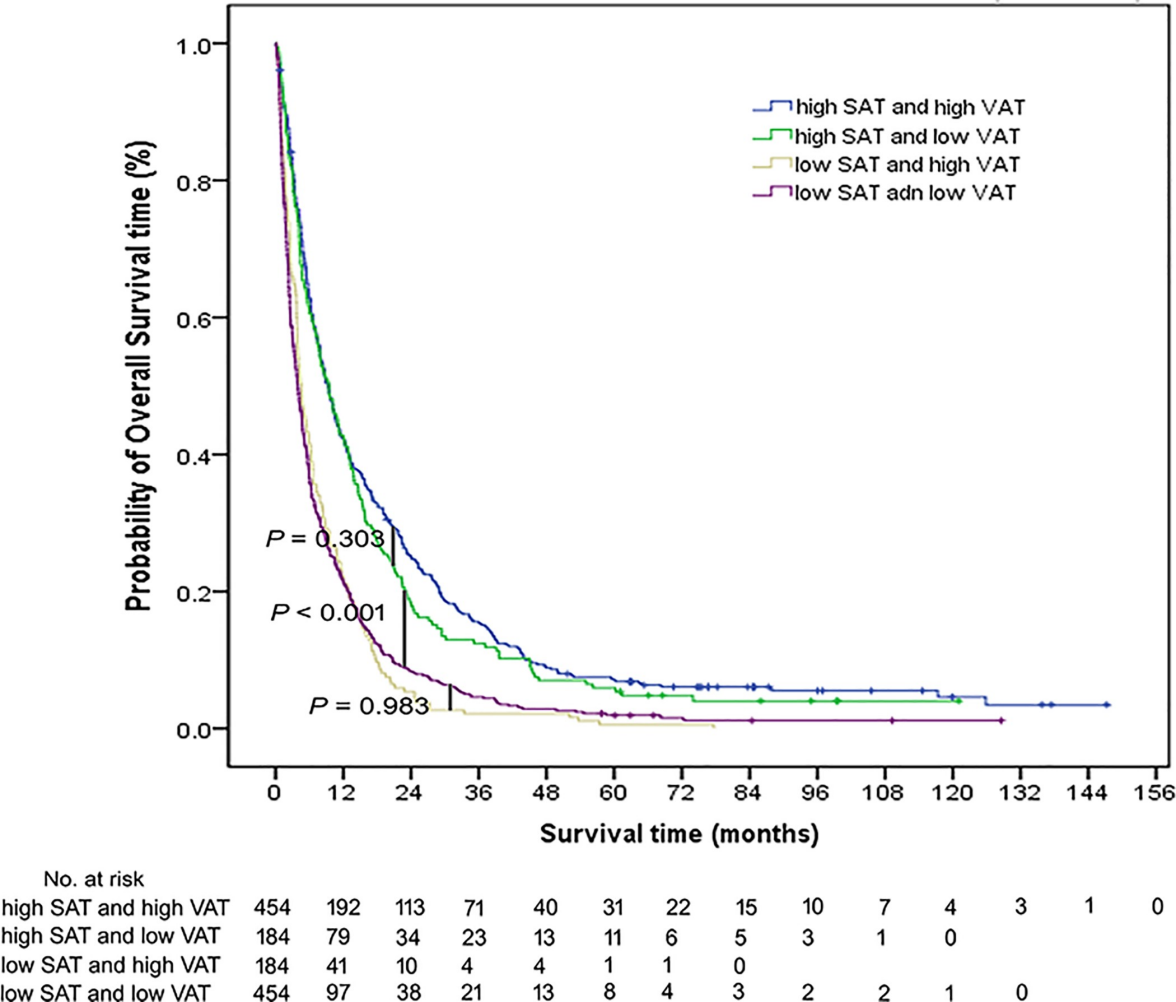
Kaplan-Meier estimates of overall survival in patients with bone metastases stratified according to subcutaneous adiposity (high *versus* low) and visceral adiposity (high *versus* low).

We subsequently examined the prognostic impact of SATI and VATI by classifying patients into high *versus* low categories. Kaplan-Meier analysis revealed no differences in OS between the high SATI/high VATI group (median survival: 9.37 months) and high SATI/low VATI group (median survival: 9.43 months; P = 0.303; Table 3). The lowest OS (3.97 months) was observed in the low SATI/low VATI group (Fig 1; Table 3).

**Table 3:** Multivariate Cox regression analysis of overall survival in patients stratified according to subcutaneous adiposity and visceral adiposity.

Subgroup	No. of patients	Median survival time (95% CI)	HR	P value
High SATI/high VATI	455	9.370 (8.116–10.624)		<0.001^a^
High SATI/low VATI	185	9.436 (7.129–11.742)	1.097 (0.920–1.307)	0.303
Low SATI/high VATI	185	4.603 (3.657–5.548)	1.882 (1.580–2.242)	<0.001^a^
Low SATI/low VATI	455	3.978 (3.362–4.594)	1.854 (1.622–2.121)	<0.001^a^

Abbreviations: CI, confidence interval; HR, hazard ratio; SATI, subcutaneous adipose tissue index; VATI, visceral adipose tissue index.

^a^Chi-square test

^b^ANOVA test.

### Prognostic stratification according to sex and body composition

**Fig 2:**
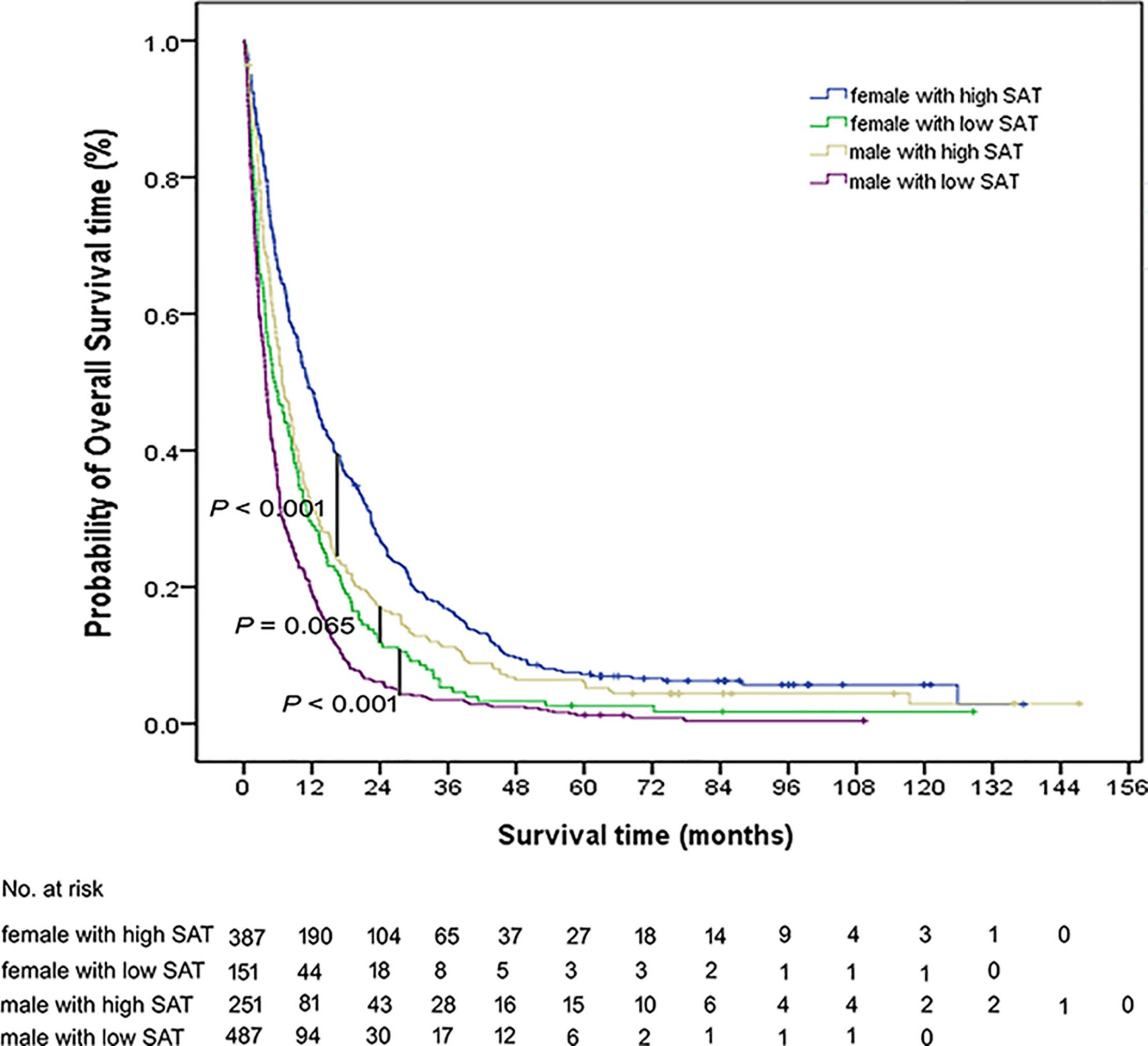
Kaplan-Meier estimates of overall survival in patients with bone metastases stratified according to subcutaneous adiposity (high *versus* low) and sex (female *versus* male).

Thereafter, both sex and SATI values were taken into account to construct four different groups. We specifically selected SATI owing to its higher prognostic value in multivariate analysis. A total of four groups were identified (male/high SATI; female/high SATI; male/low SATI; female/low SATI), with the most favorable survival figures being evident in the female/high SATI group (median OS: 11.21 months; 95% CI: 9.434−12.988 months; P<0.001 *versus* other groups). The less favorable OS survival (median: 3.847 months; 95% CI: 3.391−4.302 months) was observed in the male/low SATI group (Fig 2; Table 4).

**Table 4:** Multivariate Cox regression analysis of overall survival in patients stratified according to sex and subcutaneous adiposity.

Subgroup	No. of patients	Median survival time (95%CI)	HR	P value
Female with high SATI	388	11.211 (9.434–12.988)		<0.001^a^
Female with low SATI	152	5.293 (3.084–7.503)	1.604 (1.324–1.942)	<0.001^a^
Male with high SATI	252	6.773 (5.481–8.064)	1.312 (1.115–1.545)	<0.001^a^
Male with low SATI	488	3.847 (3.391–4.302)	2.182 (1.899–2.507)	<0.001^a^

Abbreviations: CI, confidence interval; HR, hazard ratio; SATI, subcutaneous adipose tissue index.

^a^Chi-square test

^b^ANOVA

## Discussion

The results of this retrospective analysis of prospectively collected data can be summarized as follows: 1) high SATI and VATI were independently associated with a better OS in a sample of Taiwanese patients with bone metastases, with the former showing a stronger relationship; 2) the most favorable OS was observed in women with high SATI. Although we observed associations–and not prediction or causation–our study adds to the growing literature investigating adiposity in relation to clinical outcomes of patients with malignancies.[13–15]

Currently, the association of indices of adiposity with the disease course of cancer patients remains controversial. Although adiposity seems to be positively correlated with OS in several solid tumors [16, 17], poorer survival figures have been reported for obese patients with cancer–possibly because of an increased production of growth factors and inflammatory mediators from the adipose tissue.[18] In this regard, it should be noted that adipose tissue may serve as a nutrient replacement in patients with cancer, [15, 19] but it can be also involved in tumor spread through adipokine-induced extracellular matrix remodeling.[20]

Pai et al. [21] have previously shown that SATI is strongly related to distant metastasis-free survival, locoregional control, and OS in 881 patients with head and neck cancer. Ebadi et al. [5] also demonstrated that patients with low SATI and high VATI independently predicted mortality in a sample of patients with different solid malignancies. Herein, we show that VATI, and most prominently SATI, were significantly associated with OS in Taiwanese patients with bone metastases. Controversy still exists on the relationship between VATI and clinical outcomes in patients with solid tumors.[10, 22–24] The reasons whereby SATI appears to hold a stronger association with OS over VATI in our study remain to be elucidated. However, it is notable that–differently from visceral fat (which is an active endocrine organ)–subcutaneous fat is more strictly involved in lipid and energy storage and is characterized by a lower inflammatory environment [14, 25, 26].

The study was conducted in a radiation oncology setting. Bone metastases are not only the most common site of distant spread in patients with solid malignancies but they are also the most commonly identified by radiation oncologists. The question as to whether our findings may be applied to patients with metastases to other distant sites (e.g., liver or brain) remains open. We acknowledge several limitations of the present study. First, the study was conducted in an Asian population, and it is well-known that ethnic differences exist in measures of adiposity between Asian and Caucasian populations [27]. Therefore, our findings need to be independently replicated in other geographic areas. Second, we did not segment body fat in the whole CT volume. Nonetheless, there is published evidence suggesting that measures of adiposity obtained at the L3 level through a simplified CT protocol are well-correlated to those taken at other sites [28–31]. Third, all measures of adiposity were taken in the two week preceding the start of RT. Wu et al. [32] have recently demonstrated the prognostic importance of the time at which body adiposity is assessed. However, these data were not available in this study, and we were unable to run this analysis. Finally, this was a retrospective analysis of prospectively collected data which had an exploratory nature. The application of the Bonferroni’s correction in this setting may be too conservative and was avoided. In any case, our results should be considered as preliminary and hypothesis-generating. Because we observed associations, we cannot claim any prognostic effect of adiposity indices in our population. Future longitudinal studies are required to clarify this issue further.

These limitations notwithstanding, we found that high SATI and VATI are associated with a more favorable OS in Taiwanese patients with bone metastases referred for RT. The question as to whether clinical measures aimed at improving adiposity may improve OS in this clinical population deserves further scrutiny.

## References

[1] Zhang L, Gong Z. Clinical Characteristics and Prognostic Factors in Bone Metastases from Lung Cancer. Med Sci Monit 2017;23:4087–4094 10.12659/MSM.902971 28835603 PMC5580519

[2] Riihimäki M, Hemminki A, Fallah M, Thomsen H, Sundquist K, Sundquist J, et al Metastatic sites and survival in lung cancer. Lung Cancer 2014;21:78–84 10.1016/j.lungcan.2014.07.020 25130083

[3] Coleman RE. Clinical features of metastatic bone disease and risk of skeletal morbidity. Clinical cancer research: an official journal of the American Association for Cancer Research. 2006;12(20 Pt 2):6243–6249. 10.1158/1078-0432.CCR-06-0931 17062708

[4] Zhang H, Zhu W, Biskup E, Yang W, Yang Z, Wang H, et al Incidence, risk factors and prognostic characteristics of bone metastases and skeletal-related events (SREs) in breast cancer patients: A systematic review of the real world data. J Bone Oncol 2018;3:38–50 10.1016/j.jbo.2018.01.004 PMC5832676 29511626

[5] Ebadi M, Martin L, Ghosh S, Field CJ, Lehner R, Baracos VE, et al Subcutaneous adiposity is an independent predictor of mortality in cancer patients. Br J Cancer. 2017;117(1):148–155. 10.1038/bjc.2017.149 28588319 PMC5520211

[6] Rosenquist K, Wennerberg J, Schildt EB, Bladstrom A, Goran HB, Andersson G. Oral status, oral infections and some lifestyle factors as risk factors for oral and oropharyngeal squamous cell carcinoma. A population-based case-control study in southern Sweden. Acta Otolaryngol 2005;125(12):1327–1336. 10.1080/00016480510012273 16303683

[7] Valencak TG, Osterrieder A, Schulz TJ. Sex matters: The effects of biological sex on adipose tissue biology and energy metabolism. Redox Biol 2017;12:806–813. 10.1016/j.redox.2017.04.012 28441629 PMC5406544

[8] Lee HW, Jeong BC, Seo SI, Jeon SS, Lee HM, Choi HY, et al Prognostic significance of visceral obesity in patients with advanced renal cell carcinoma undergoing nephrectomy. Int J Urol 2015;22(5):455–461. 10.1111/iju.12716 25631365

[9] Fujiwara N, Nakagawa H, Kudo Y, Tateishi R, Taguri M, Watadani T, et al Sarcopenia, intramuscular fat deposition, and visceral adiposity independently predict the outcomes of hepatocellular carcinoma. J Hepatol 2015;63:131–140 10.1016/j.jhep.2015.02.031 25724366

[10] Otunctemur A, Dursun M, Ozer K, Horsanali O, Ozbek E. Renal Cell Carcinoma and Visceral Adipose Index: a new risk parameter. Int Braz J Urol 2016;42(5):955–959. 10.1590/S1677-5538.IBJU.2015.0396 27532115 PMC5066891

[11] Chou YC, Lin CY, Pai PC, Tseng CK, Hsieh CE, Chang KP, et al Dose-escalated radiation therapy is associated with better overall survival in patients with bone metastases from solid tumors: a propensity score-matched study. Cancer Med 2017;6(9):2087–2097. 10.1002/cam4.1150 28809463 PMC5603838

[12] Mourtzakis M, Prado CM, Lieffers JR, Reiman T, Mccargar LJ, Baracos VE. A practical and precise approach to quantification of body composition in cancer patients using computed tomography images acquired during routine care. Appl Physiol Nutr Metab 2008;33(5):997–1006. 10.1139/H08-075 18923576

[13] Tsang NM, Pai PC, Chuang CC, Chuang WC, Tseng CK, Chang KP, et al Overweight and obesity predict better overall survival rates in cancer patients with distant metastases. Cancer Med 2016;5(4):665–675. 10.1002/cam4.634 26811258 PMC4831285

[14] Bays H. Central obesity as a clinical marker of adiposopathy; increased visceral adiposity as a surrogate marker for global fat dysfunction. Curr Opin Endocrinol Diabetes Obes 2014;21:345–351 10.1097/MED.0000000000000093 25106000 PMC4154790

[15] Lennon H, Sperrin M, Badrick E, Renehan AG. The Obesity Paradox in Cancer: a Review. Curr Oncol Rep 2016;18(9):56 10.1007/s11912-016-0539-4 27475805 PMC4967417

[16] Antoun S, Bayar A, Ileana E, Laplanche A, Fizazi K, Di Palma M, et al High subcutaneous adipose tissue predicts the prognosis in metastatic castration-resistant prostate cancer patients in post chemotherapy setting. Eur J Cancer 2015;51:2570–2577 10.1016/j.ejca.2015.07.042 26278649

[17] Ladoire S, Bonnetain F, Gauthier M, Zanetta S, Petit JM, Guiu S, et al Visceral Fat Area as a New Independent Predictive Factor of Survival in Patients with Metastatic Renal Cell Carcinoma Treated with Antiangiogenic Agents. Oncologist 2011;16:71–81 10.1634/theoncologist.2010-0227 21212435 PMC3228050

[18] Cozzo AJ, Fuller AM, Makowski L. Contribution of Adipose Tissue to Development of Cancer. Compr Physiol 2017;8(1):237–282. 10.1002/cphy.c170008 29357128 PMC5806627

[19] Demark-Wahnefried W, Platz EA, Ligibel JA, Blair CK, Courneya KS, Meyerhardt JA, et al The role of obesity in cancer survival and recurrence. Cancer Epidemiol Biomarkers Prev 2012;21(8):1244–1259. 10.1158/1055-9965.EPI-12-0485 22695735 PMC3415558

[20] Oudin MJ, Jonas O, Kosciuk T, Broye LC, Guido BC, Wyckoff J, et al Tumor Cell-Driven Extracellular Matrix Remodeling Drives Haptotaxis during Metastatic Progression. Cancer Discov 2016;6(5):516–531. 10.1158/2159-8290.CD-15-1183 26811325 PMC4854754

[21] Pai PC, Chuang CC, Chuang WC, Tsang NM, Tseng CK, Chen KH, et al Pretreatment subcutaneous adipose tissue predicts the outcomes of patients with head and neck cancer receiving definitive radiation and chemoradiation in Taiwan. Cancer Med 2018;7(5):1630–1641. 10.1002/cam4.1365 29608254 PMC5943483

[22] Grignol VP, Smith AD, Shlapak D, Zhang X, Del Campo SM, Carson WE. Increased visceral to subcutaneous fat ratio is associated with decreased overall survival in patients with metastatic melanoma receiving anti-angiogenic therapy. Surg Oncol 2015;24(4):353–358. 10.1016/j.suronc.2015.09.002 26690825 PMC6586412

[23] Lee KH, Kang BK, Ahn BK. Higher visceral fat area/subcutaneous fat area ratio measured by computed tomography is associated with recurrence and poor survival in patients with mid and low rectal cancers. Int J Colorectal Dis 2018;33:1303–1307. 10.1007/s00384-018-3065-z 29713823

[24] Harada K, Baba Y, Ishimoto T, Kosumi K, Tokunaga R, Izumi D, et al Low Visceral Fat Content is Associated with Poor Prognosis in a Database of 507 Upper Gastrointestinal Cancers. Ann Surg Oncol 2015;22:3946–3953 10.1245/s10434-015-4432-4 25712800

[25] Mccarty MF. Modulation of adipocyte lipoprotein lipase expression as a strategy for preventing or treating visceral obesity. Med hypotheses 2001;57(2):192–200. 10.1054/mehy.2001.1317 11461172

[26] Christen T, Sheikine Y, Rocha VZ, Hurwitz S, Goldfine AB, Di Carli M, et al Increased glucose uptake in visceral versus subcutaneous adipose tissue revealed by PET imaging. JACC Cardiovasc imaging 2010;3(8):843–851. 10.1016/j.jcmg.2010.06.004 20705265 PMC4042675

[27] Heymsfield SB, Peterson CM, Thomas DM, Heo M, Schuna JM Jr. Why are there race/ethnic differences in adult body mass index-adiposity relationships? A quantitative critical review. Obes Rev 2016;17(3):262–75. 10.1111/obr.12358 26663309 PMC4968570

[28] Shen W, Punyanitya M, Wang Z, Gallagher D, St-Onge MP, Albu J, et al Visceral adipose tissue: relations between single-slice areas and total volume. Am J Clin Nutr 2004;80:271–278. 10.1093/ajcn/80.2.271 . 15277145 PMC2040041

[29] Noumura Y, Kamishima T, Sutherland K, Nishimura H. Visceral adipose tissue area measurement at a single level: can it represent visceral adipose tissue volume? Br J Radiol 2017;90:20170253 10.1259/bjr.20170253 28707539 PMC5858805

[30] Schweitzer L, Geisler C, Pourhassan M, Braun W, Gluer CC, Bosy-Westphal A, et al What is the best reference site for a single MRI slice to assess whole-body skeletal muscle and adipose tissue volumes in healthy adults? Am J Clin Nutr 2015;102:58–65. 10.3945/ajcn.115.111203 26016860

[31] Demerath EW, Shen W, Lee M, Choh AC, Czerwinski SA, Siervogel RM, Towne B. Approximation of total visceral adipose tissue with a single magnetic resonance image. Am J Clin Nutr 2007;85:362–368. 10.1093/ajcn/85.2.362 17284730 PMC2883309

[32] Wu S, Liu J, Wang X, Li M, Gan Y, Tang Y. Association of obesity and overweight with overall survival in colorectal cancer patients: a meta-analysis of 29 studies. Cancer Causes Control. 2014;25:1489–1502. 10.1007/s10552-014-0450-y 25070668

