# Supplementary material for: Association of subcutaneous and visceral adipose tissue with overall survival in Taiwanese patients with bone metastases – results from a retrospective analysis of consecutively collected data
Source: PLoS One. 2020 Jan 30;15(1):e0228360. doi: 10.1371/journal.pone.0228360 (PMC6992206; doi:10.1371/journal.pone.0228360)
Supplement: Attachment — Submitted filename: Response to Reviewers.doc [file pone.0228360.s001.doc]

**AUTHORS’ REPLY TO REVIEWER’S #1 COMMENTS**

**1. The association between body fat and survival in many cancers is well-known and established. Hence, the study does not add much new to the current knowledge. However, I acknowledge that this is not necessarily relevant for PLoS ONE, so this point may be disregarded by the editor.**

REPLY. We thank the Reviewer for the constructive criticism. We concur that the association between adiposity and survival in patients with malignancies has been extensively investigated in the past. However, this study may represent a valid addition to the existing literature for the following reasons: 1) the association of subcutaneous adiposity and visceral adiposity with survival endpoints in patients with cancer remains controversial, 2) we specifically focused on patients with bone metastases who were referred for radiotherapy (see below for further clarifications on study population); 3) the study was conducted in an Asian population (it is well-known that ethnic differences exist in measures of adiposity between Asian and Caucasian populations). We believe that these points should be considered when dealing with the novelty of our work. Please note that the title has been revised to highlight the ethnic origin of the study population.

**The study rationale is not quite clear. Why focus on patients with bone metastases? Other metastases also herald poor prognosis (liver, brain ...).**

REPLY. We are grateful to the Reviewer for the cogent comment. We specifically focused on bone metastases for the following reasons. First, the study was conducted in a radiation oncology setting. Bone metastases are not only the most common site of distant spread in patients with solid malignancies but they are also the most commonly identified by radiation oncologists. We did focus on bone metastases not only because they portend a poor prognosis, but also in light of their high frequency. Second, patients with bone metastases commonly present with pain and an impaired quality of life, ultimately requiring referral for radiotherapy. We therefore believe that our study population is of special interest for radiation therapists. We nonetheless acknowledged the lack of inclusion of patients with metastases at other sites as a limitation inherent in our study (please see the revised “Discussion” section).

**The chance was missed to segment the body fat in the whole CT scan volume and thus provide a more accurate estimate.**

REPLY. We concur with the Reviewer that whole-body computed tomography (CT) can actually provide a more accurate and comprehensive estimation of adiposity. However, there is published evidence (see references 1−4 below) suggesting that measures of adiposity obtained at the L3 level through a simplified CT protocol are well-correlated to those taken at other sites. We are aware that this is a potential limitation inherent in our study, which has been acknowledged in the revised “Discussion” section.

**A biostatistician should be consulted for the statistical analysis, which is not well done (the current analysis leads to spuriously low p-values).**

REPLY. This study was designed as a retrospective analysis of prospectively collected data (a point which has been clarified in revised paper). The statistical approach was in line with the analyses conducted in other published papers from our group (see references 5−8 below). With regard to the low p-values, they may stem from a multiple comparison problem. However, the application of the Bonferroni’s correction may be too conservative in an exploratory analysis. We nonetheless believe that this is a potential caveat inherent in our study, which has been addressed in the revised “Discussion” section.

**5. The term "predictor" should be avoided throughout the manuscript. In a retrospective study, one can only find associations. Predictions can only be made with a prospective trial. This leads me to the next point**

REPLY. We thank the Reviewer for the pertinent observation. In the revised version of the paper, we made appropriate revisions to highlight that we observed associations, not prediction or causation. We also acknowledged the retrospective design as a major study limitation in the “Discussion” section.

**6. The authors grossly overinterpret the results of their study. Although this is unfortunately the norm in many scientific journals where authors need to "sell" their study, per PLoS ONE policy this is not needed. To give the authors an example: "More importantly, our findings may pave the way for aggressive therapeutic interventions in the subset of patients who are expected to have more favorable survival figures" To make such a statement, a prospective randomised clinical trial is needed.**

REPLY. The Reviewer is entirely right and we apologize for the excessive emphasis put on the significance of our findings. In the revised version of the paper, several statements were toned down, the main limitations were highlighted, and the conclusions were drawn more prudently. We have highlighted these points as a relevant future research topic in the revised “Discussion” section.

*References*

1. Shen W, Punyanitya M, Wang Z, Gallagher D, St-Onge MP, Albu J, Heymsfield SB, Heshka S. Visceral adipose tissue: relations between single-slice areas and total volume. Am J Clin Nutr 2004;80:271–278.

2. Noumura Y, Kamishima T, Sutherland K, Nishimura H. Visceral adipose tissue area measurement at a single level: can it represent visceral adipose tissue volume? Br J Radiol 2017;90:20170253.

3. Schweitzer L, Geisler C, Pourhassan M, Braun W, Gluer CC, Bosy- Westphal A, Muller MJ. What is the best reference site for a single MRI slice to assess whole-body skeletal muscle and adipose tissue volumes in healthy adults? Am J Clin Nutr 2015;102:58–65.

4. Demerath EW, Shen W, Lee M, Choh AC, Czerwinski SA, Siervogel RM, Towne B. Approximation of total visceral adipose tissue with a single magnetic resonance image. Am J Clin Nutr 2007;85:362-368.

5. Tsang NM, Pai PC, Chuang CC, Chuang WC, Tseng CK, Chang KP, Yen TC, Lin JD, Chang JT. Overweight and obesity predict better overall survival rates in cancer patients with distant metastases. Cancer Med 2016;5(4):665–675.

6. Pai PC, Chuang CC, Chuang WC, Tsang NM, Tseng CK, Chen KH, et al. Pretreatment subcutaneous adipose tissue predicts the outcomes of patients with head and neck cancer receiving definitive radiation and chemoradiation in Taiwan. Cancer Med. 2018;7:1630–1641.

7. Pai PC, Chuang CC, Tseng CK, Tsang NM, Chang KP, Yen TC, et al. Impact of pretreatment body mass index on patients with head-and-neck cancer treated with radiation. Int J Radiat Oncol Biol Phys. 2012;83:e93-e100.

8. Tsang NM, Chuang CC, Tseng CK, Hao SP, Kuo TT, Lin CY et al. Presence of the latent membrane protein 1 gene in nasopharyngeal swabs from patients with mucosal recurrent nasopharyngeal carcinoma. Cancer 2003;98:2385–2392.

**AUTHORS’ REPLY TO REVIEWER’S #2 COMMENTS**

**On page 25, the authors report as a limitation of this study: "Body composition was assessed in all patients prior to the first RT session and not when bone metastases were diagnosed." However, they do not clearly explain how this may imply the results found. Thus, I suggest to the authors, as a minor review, to further clarify this issue, given that the time when BMI was determined is relevant to the observed patterns of association.
We suggest the authors review the World Cancer Research Fund (WCRF) report on the effect of risk factors on survival among women with breast cancer. This recent report added a very useful classification—namely, determination of BMI either at pre-, peri-, or post-diagnosis (the later typically 12 months after the initial treatment) of câncer. From these, different patterns of associations emerge. In a meta-analysis of 29 studies evaluating the impact of BMI on survival in patients with colorectal cancer, Wu et al. observed that increasing pre-diagnosis BMI prognosticated for a poor survival but that post-treatment overweight was associated with improved survival, i.e., the obesity paradox.

Suggested References to Authors:

WCRF. World Cancer Research Fund International. Continuous Update Project Report: diet, nutrition, physical activity, and breast cancer survivors. 2014. Available at:www.wcrf.org/sites/default/files/Breast-Cancer-Survivors-2014-Report.pdf [Accessed 20th Dec 2014]. 2014.

Wu S, Liu J, Wang X, Li M, Gan Y, Tang Y. Association of obesity and overweight with overall survival in colorectal cancer patients: a meta-analysis of 29 studies. Cancer Causes Control. 2014;25:1489–1502. doi: 10.1007/s10552-014-0450-y.**

REPLY. We thank the Reviewer for the constructive observations. In the revised version of the paper, we clarified that all measures of adiposity were taken in the two week preceding the start of RT. With regard to the World Cancer Research Fund (WCRF) report and the paper by Wu et al., we realize the prognostic importance of the time at which body adiposity is assessed. However, these data were not available in this study, and we were therefore unable to run this analysis. We have highlighted this point as a relevant future research topic in the revised “Discussion” section.

We had mentioned in the Discussion line 256: “Fourth, all measures of adiposity were taken in the two week preceding the start of RT. Wu et al. (32) have recently demonstrated the prognostic importance of the time at which body adiposity is assessed. However, the data of post-diagnosis of cancer (12 months after initial treatment) were not available in this study, and we were unable to run this analysis. ”

**AUTHORS’ REPLY TO REVIEWER’S #3 COMMENTS**

**I consider the piece is valuable. However, results must be explained more in detail to be able to trace interpretations offered at discussion. Manuscript should be reviewed for grammatical errors (for example patient demographics, should be more in relation to the concept assessment than the way variables were implemented), specially for critical interpretations of data and analyses. Recommendations for more aggressive therapeutics is not included in the analysis, but is being offered as a conclusion, therefore I recommend to review this issue and explain more about how this is derived from the analysis.**

REPLY. We thank the Reviewer for the constructive criticisms. In order to address the concern, the following changes were implemented: 1) the paper has been revised for style and presentation; 2) the nature of the analysis was clarified (retrospective analysis of prospectively collected data) and any reference to prediction or causation were removed; 3) we removed the emphasis on therapeutic recommendations because we realize that they are unwarranted based on our current data.
